# Reply to Sacco: Complete spaces as outcomes of evolutionary optimization

**DOI:** 10.1073/pnas.2605961123

**Published:** 2026-03-19

**Authors:** Philip LaPorte, Christian Hilbe, Nikoleta E. Glynatsi, Martin A. Nowak

**Affiliations:** ^a^Department of Mathematics, University of California, Berkeley, CA 94720; ^b^Department of Mathematics, Harvard University, Cambridge, MA 02138; ^c^Research Group on the Dynamics of Social Behavior, Interdisciplinary Transformation University, Linz A-4040, Austria; ^d^Discrete Event Simulation Research Team, RIKEN Center for Computational Science, Kobe 650-0047, Japan

We thank Sacco (1) for thoughtful commentary on our work (2). In our paper, we address a completeness property of certain strategy spaces: The strategies they contain have best replies in the space, and payoffs achievable by opponents outside of the space can be achieved within the space.

Sacco suggests interpreting strategy spaces as cognitive architectures. For example, an agent may be hardwired to have only one round of memory. They can only implement memory-1 strategies. Natural selection acts on these cognitive architectures. Sacco makes an interesting argument that we can expect completeness as an outcome of evolutionary optimization.

The argument appeals to a tradeoff between computational efficiency and strategic effectiveness. Complete spaces can achieve both.

Computational efficiency comes from two sources. First, the strategies in any restricted space have a limited number of parameters. Second, the recursiveness property, which implies completeness, means that executing such a strategy requires only incremental memory updating at each step.

Strategic effectiveness, Sacco suggests, is implied by the completeness property itself.

For the latter point, we believe that further investigation could be very useful. Although strategies in complete spaces have best replies of the same kind, they may not necessarily achieve high payoff against some ensembles of strategies.

What can be said is that when a homogeneous population reaches strategic equilibrium within a complete space, more complicated mutants outside of the space face selective disadvantage. They are computationally inefficient with no strategic benefit.

In that sense, complete spaces may indeed represent stable points of cognitive evolution.

In general, the relative fitness of various strategy spaces (3) is a fascinating topic for further study. In our opinion, completeness is one relevant factor in this fitness landscape. Additional work could, for example, study the extent to which a strategy of one type can be a best reply to a wide range of opponent strategies.

Overall, we are enthusiastic about the potential for further discoveries on the evolution of strategic complexity and how it relates to completeness.
